# RNF25 serves as a novel diagnostic and prognostic biomarker in multiple myeloma: a multi-cohort integrative analysis

**DOI:** 10.1186/s41065-025-00631-0

**Published:** 2025-12-28

**Authors:** Bowen Jiang, Qiuyue An, Lunbi Wu, Xinyi Zhang, Hongxin Xu, Suliang Wang, Yanzhang Qu

**Affiliations:** 1 The Fourth Department of Hematology, The Second Affiliated Hospital of Qiqihar Medical University, No. 64 Zhonghua West Road, Jianhua District, Qiqihar, 161000 China; 2 Department of Infectious Disease, The Second Affiliated Hospital of Qiqihar Medical University, Qiqihar, 161000 China; 3 Department of Laboratory Diagnosis, The Second Affiliated Hospital of Qiqihar Medical University, Qiqihar, 161000 China; 4 Department of Rheumatology and Immunology, The Second Affiliated Hospital of Qiqihar Medical University, Qiqihar, 161000 China

**Keywords:** Multiple myeloma, RNF25, Ubiquitination, Prognostic biomarker, Immune microenvironment

## Abstract

**Background:**

Ubiquitination-related genes (UbRGs) play critical roles in tumor biology. However, their functions in multiple myeloma (MM) remain insufficiently explored.

**Methods:**

Transcriptomic data from public databases were integrated to identify UbRGs in MM through WGCNA, differential expression, and protein–protein interaction (PPI) analyses. Functional roles were examined by GO, KEGG, and GSEA, while immune infiltration and drug sensitivity were evaluated using CIBERSORT, ESTIMATE, and OncoPredict. UbRG expression was validated in clinical samples and cell lines by qRT-PCR and western blot, and functional effects of UbRG knockdown in MM cells were examined using CCK-8, Transwell, EdU, and TUNEL assays.

**Results:**

Ring Finger Protein 25 (RNF25) is a ubiquitination-related gene closely associated with MM, exhibiting consistent overexpression across multiple independent datasets. Elevated RNF25 expression is significantly correlated with poorer overall survival and serves as an independent adverse prognostic factor. Functional enrichment analysis revealed that RNF25 may regulate key pathways such as ATP-dependent chromatin remodeling, cell cycle progression, and ubiquitin-mediated proteolysis. Immune analysis further indicated that high RNF25 expression is associated with reduced immune and stromal scores, increased plasma cell infiltration, and elevated T cell co-inhibition activity. RNF25 was highly expressed in tumor tissues from clinical samples, and its knockdown significantly reduced the viability, proliferation, and migration of U266 cells, while promoting apoptosis.

**Conclusions:**

RNF25 may serve as a potential prognostic biomarker for MM and holds promise as a candidate therapeutic target.

**Supplementary Information:**

The online version contains supplementary material available at 10.1186/s41065-025-00631-0.

## Introduction

Multiple myeloma (MM) is a hematological malignancy originating from plasma cells, characterized by clonal proliferation and marked heterogeneity. Its pathogenesis is complex, and clinical manifestations are highly variable [1]. MM is typified by the aberrant accumulation of malignant plasma cells in the bone marrow, accompanied by excessive production of monoclonal immunoglobulins [2, 3]. These abnormalities lead to multi-organ dysfunction, including impaired hematopoiesis, extensive bone destruction, renal insufficiency, hypercalcemia, and immunosuppression [4]. Globally, the incidence of MM continues to rise, making it a significant threat to public health [5].

Over the past decades, substantial advances in MM treatment have markedly improved patient outcomes and quality of life [6]. While conventional chemotherapy and radiotherapy remain foundational therapeutic approaches, newer modalities—such as proteasome inhibitors (PIs), immunomodulatory drugs (IMiDs), and monoclonal antibodies—have demonstrated promising efficacy and are now integrated into standard care regimens [7]. The combination of high-dose chemotherapy, tandem hematopoietic stem cell transplantation, and the application of PIs and IMiDs has enabled long-term survival for many patients, with approximately one-third potentially achieving a cure [8]. Nevertheless, treatment-related adverse events (AEs), drug resistance, and disease relapse remain major challenges, highlighting the urgent need to elucidate novel pathogenic mechanisms and therapeutic targets at the molecular level.

The ubiquitin–proteasome system (UPS) is the primary intracellular mechanism for protein degradation and plays a central role in maintaining protein homeostasis and regulating various biological processes, including signal transduction, cell cycle progression, DNA repair, and epigenetic modifications [9]. In MM, dysregulation of the UPS is considered one of the key drivers of tumor initiation and progression. Due to the high levels of immunoglobulin synthesis and the consequent accumulation of misfolded proteins, MM cells are highly dependent on proteostasis and therefore particularly sensitive to UPS dysfunction [10]. Based on this biological vulnerability, inhibition of the UPS—especially proteasomal activity—has become a crucial therapeutic strategy for MM [10]. In addition to mediating protein degradation, the UPS regulates several key signaling pathways involved in the pathogenesis of MM. Among these, the NF-κB signaling pathway is essential for maintaining MM cell survival and resistance to apoptosis, and its activation is precisely regulated by ubiquitination processes [11]. Furthermore, studies have shown that the E3 ubiquitin ligase BMI1 is significantly upregulated in MM, where it promotes cell proliferation by regulating histone ubiquitination and enhances MM cell growth both in vivo and in vitro [12].

Although various UPS-targeted therapies have been successfully introduced into clinical practice and demonstrated favorable outcomes, challenges such as declining efficacy, adverse effects, and acquired resistance remain [13]. Therefore, systematic identification of key ubiquitination-related genes (UbRGs) in MM and clarification of their roles in disease initiation, progression, and prognosis may provide new insights for precision diagnosis and therapy of MM.

This study aimed to integrate transcriptomic data from the TCGA and GEO databases, combined with comprehensive bioinformatics analyses, to identify key UbRGs associated with MM. Their functional roles and prognostic significance in MM were further explored to uncover potential therapeutic targets.

## Materials and methods

### Data sources

In this study, transcriptomic data and corresponding clinical information from the TCGA dataset (MMRF-CoMMpass; 838 MM samples) were integrated. Additionally, gene expression profiles were retrieved from the GEO database, including GSE118985 (68 healthy controls and 460 newly diagnosed MM patients), GSE6477 (15 healthy controls and 73 newly diagnosed MM patients), and GSE4581 (414 MM samples with survival data). A total of 1,380 UbRGs were retrieved from the iUUCD 2.0 database, and after removing duplicate gene names, 1,367 unique UbRGs were retained for subsequent analyses (Table S1).

### Weighted gene Co-expression network analysis (WGCNA)

Based on the transcriptomic data from GSE118985, a weighted gene co-expression network was constructed using the WGCNA package in R (version 1.72–5.72) [14]. Genes with log_2_TPM ≥ 1 were included, and Pearson correlation coefficients were calculated to assess gene pairwise relationships. An optimal soft-thresholding power (β) was selected to generate an adjacency matrix adhering to scale-free topology. This matrix was subsequently transformed into a topological overlap matrix (TOM), followed by hierarchical clustering to identify gene modules. Module significance and gene significance were computed to identify modules strongly associated with disease phenotypes, from which core genes were extracted.

### Differential expression analysis

Differentially expressed genes (DEGs) between groups were identified using the limma package in R (version 3.62.2) [15]. Genes with |log_2_ fold change (FC)| > 0.5 and a false discovery rate (FDR) < 0.05 were considered significantly differentially expressed.

### Functional enrichment analysis

The clusterProfiler package (v4.14.6) was employed to perform Gene Ontology (GO) enrichment analysis, including Biological Process (BP), Molecular Function (MF), and Cellular Component (CC), as well as Kyoto Encyclopedia of Genes and Genomes (KEGG) pathway analysis [16], with significance set at p.adjust < 0.05. Gene Set Enrichment Analysis (GSEA) was further carried out by dividing samples into high- and low-expression groups according to the median expression of target genes to identify enriched pathways (p.adjust < 0.05).

### Protein–Protein interaction (PPI) network and hub gene identification

PPI networks were constructed using the STRING database (https://string-db.org/) based on combined scores of predicted interactions. Cytoscape software was used for network visualization, and topological characteristics were assessed using four algorithms—Density of Maximum Neighborhood Component (DMNC), BottleNeck, Betweenness, and ClusteringCoefficient—to identify the top 10 hub genes. The intersection of these results was considered the final hub gene set.

### Immune-Related analysis

The CIBERSORT algorithm [17] was used to estimate the relative abundance of immune cell subtypes within each sample. Additionally, single-sample GSEA (ssGSEA) was performed using the GSVA package (version 2.0.6) to quantify immune function activity based on 13 predefined gene sets [18]. Immune Score, Stromal Score, and ESTIMATE Score were calculated using the estimate package (version 1.0.13).

### Survival analysis and prognostic model construction

Kaplan–Meier analysis was conducted with the survival (v3.8) and survminer (v0.5.0) packages in R, and Cox proportional hazards models were used to assess the prognostic value of hub genes. Model performance was evaluated by receiver operating characteristic (ROC) curves and area under the curve (AUC) generated with the pROC package (v1.18.5).

### Drug sensitivity prediction

Drug sensitivity was predicted using the oncoPredict package (version 1.2) [19] in R, based on half-maximal inhibitory concentration (IC_50_) values from the GDSC V2 database.

### Molecular Docking

The 3D structure of Temozolomide (CID: 5394) was downloaded from the PubChem database, and the target protein structure was obtained from the AlphaFold database (ID: AF-Q96BH1-F1-v4). Ligand and receptor preprocessing was conducted using Discovery Studio 4.5. 5 potential binding cavities of protein were identified using the CB-DOCK2 server. Molecular docking was then performed using the FitDock protocol within AutoDock Vina, and the optimal binding mode was visualized in 2D and 3D using Discovery Studio.

### Molecular dynamics simulation

Ten-nanosecond molecular dynamics simulations were conducted using Gromacs (version 2024.4). The CHARMM36 force field and TIP3P water model were employed. The topology of the receptor–ligand complex was processed and ionized for system neutrality. The simulation protocol included energy minimization, followed by NVT and NPT equilibration phases. Temperature and pressure were maintained at 300 K and 1 bar, respectively. After simulation, trajectories were aligned and periodic boundary artifacts removed. Root-mean-square deviation (RMSD), root-mean-square fluctuation (RMSF), radius of gyration (Rg), and hydrogen bond dynamics were calculated and visualized.

### Clinical samples

Fifteen pairs of MM tissues and the corresponding adjacent normal tissues were collected from MM patients at the Second Affiliated Hospital of Qiqihar Medical University. The study protocol was approved by the Ethics Committee of the Second Affiliated Hospital of Qiqihar Medical University (approval number: (Qi) ethics approval [2024] No. 86) in accordance with the Declaration of Helsinki, and written informed consent was obtained from all participants.

All clinical samples in this study were obtained from bone marrow aspirates and classified based on bone marrow morphological examination. The diagnosis of MM in the experimental group followed international criteria, requiring a bone marrow clonal plasma cell proportion ≥ 10%, together with at least one myeloma-defining event, including CRAB features (hypercalcemia, renal insufficiency, anemia, or radiologically confirmed osteolytic lesions) or SLiM biomarkers (bone marrow clonal plasma cells ≥ 60%, involved/uninvolved serum free light-chain ratio ≥ 100, or > 1 focal lesion ≥ 5 mm detected by MRI). The control group consisted of patients with iron-deficiency anemia, diagnosed based on bone marrow iron staining showing the absence of stainable iron and < 15% sideroblasts [20].

### Cell culture and transfection

Human immortalized B-lymphoblastoid cell lines (B-LCLs, JY-J14201, Jinyuan Biotechnology) were cultured in RPMI-1640 medium supplemented with 10% fetal bovine serum (FBS; Gibco, USA) and 1% penicillin–streptomycin (Invitrogen, USA) at 37 °C in a humidified atmosphere containing 5% CO_2_. MM cell lines MM.1 S (BNCC270394, BeNa) and U266 (BNCC342290, BeNa) were maintained in RPMI-1640 medium supplemented with 10% FBS and 1% penicillin–streptomycin under the same culture conditions.

For overexpression of RNF25 in U266 cells, cells were transfected with pcDNA3.1 negative control (OE-NC) and pcDNA3.1- RNF25 (OE-RNF25) plasmids using Lipofectamine 2000 reagent (Invitrogen, USA). For gene silencing experiments, U266 cells were transfected with small interfering RNAs (siRNAs) specifically targeting RNF25 (si-RNF25-1/2/3) using Lipofectamine 2000 reagent according to the manufacturer’s instructions. The sequences of siRNAs were as follows: si-RNF25-1, 5’-GAGGACCAGGATTCAGTAT-3’; si-RNF25-2, 5’-GATGAACTACAGGATTAAA-3’; si-RNF25-3, 5’-TGGAGGGGGGCAATAAAGAT-3’. The control group received no treatment.

### Cell viability assay

U266 cells (2 × 10^3^/well) were plated in 96-well plates and maintained at 37 °C with 5% CO_2_. At 24, 48, and 72 h, cell viability was determined using the CCK-8 assay (C0039, Beyotime). Following 1 h incubation with the reagent, absorbance at 450 nm was recorded using a microplate reader (DNM-9602, Perlong).

### Transwell assay

After serum starvation of U266 cells for 12–24 h, the cell density was adjusted to 5 × 10^5^/mL with serum-free medium containing BSA, and 200 µL of the suspension was seeded into the upper chamber. The lower chamber was filled with 500 µL complete medium containing FBS. Following 24 h incubation at 37 °C with 5% CO_2_, migrated cells were fixed with 500 µL cell fixation solution (BN20094, Biorigin) and stained with 0.1% crystal violet. Images were captured under a light microscope.

### EdU staining assay

Cell proliferation was evaluated using an EdU assay kit (CA1174, Solarbio). Cells were fixed with 4% paraformaldehyde, permeabilized with 0.3% Triton X-100, stained with the Click reaction reagent, counterstained with Hoechst 33,342, and visualized by fluorescence microscopy.

### TUNEL staining assay

Apoptosis was detected using a TUNEL assay kit (BN16013, Bioorigin). Cells were fixed with 4% paraformaldehyde, permeabilized with 0.1% Triton X-100, treated with the TUNEL reaction mixture, and counterstained with DAPI before fluorescence microscopy.

### Quantitative reverse transcription PCR (qRT-PCR)

Total RNA from cells was extracted using TriQuick Reagent (R1100, Solarbio), whereas total RNA from tissue samples was extracted using the Redzol method. Approximately 10 mg of tissue was homogenized in 0.1 mL Redzol and incubated at room temperature for 5 min to ensure complete lysis. Chloroform was added at a ratio of 0.02 mL per 0.1 mL of Redzol, the mixture was vigorously shaken, and incubated for 2–3 min at room temperature, followed by centrifugation at 12,000 × g for 15 min at 4 °C. The upper aqueous phase was carefully transferred to a new tube. RNA was precipitated by adding 50 µL isopropanol, mixing thoroughly, and incubating at room temperature for 10 min, then centrifuging at 12,000 × g for 10 min at 4 °C. The pellet was washed 2–3 times with 75% ethanol (7,500 × g, 5 min, 4 °C), air-dried for 5–10 min, and dissolved in 50 µL RNase-free water, followed by incubation at 55–60 °C for 10 min to facilitate complete dissolution. A 2-µL aliquot was used for RNA quantification, and the remaining RNA was stored at − 80 °C.

Total RNA was reverse transcribed into cDNA with the SureScript™ First-Strand cDNA Synthesis Kit (QP056, GeneCopoeia). qRT-PCR was performed using 2× SYBR Green qPCR Master Mix (NoneROX) (G3320-05, Servicebio) on a real-time PCR system. GAPDH served as the internal control, and relative gene expression was calculated using the 2^^–ΔΔCt^ method. The primer sequences were as follows: GAPDH, forward 5’-CAGGAGCGAGACCCCACTAA-3’ and reverse 5’-ATCACGCCACAGCTTTCCAG-3’; RNF25, forward 5’-AACATCCCTCATGGCCAGTG-3’and reverse 5’-TGCTGATTTGACCTGGTCCC-3’.

### Western blotting

Total protein was extracted with RIPA buffer (BN25011-A, Bioorigin) and quantified via BCA assay (SL201, UtiBody). Proteins were incubated overnight at 4 °C with anti-RNF25 (24536-1-AP, proteintech) primary antibody, followed by incubation of Goat Anti-Rabbit IgG H&L (HRP) (ab205718, Abcam) secondary antibody for 1 h at room temperature. Bands were visualized using ultrasensitive chemiluminescence detection kit (BN16009, Bioorigin) and imaged with the ChemiScope6100 imaging system. Densitometry was performed using ImageJ.

### Statistical analysis

All statistical analyses were performed in R software (version 4.4.1) and GraphPad Prism 9.41. For experimental data, the Student’s t-test was used for comparisons between two groups, while one-way analysis of variance (ANOVA) was applied for comparisons among three or more groups. Data are shown as mean ± SD, with *p* < 0.05 considered significant.

## Results

### Identification of Ubiquitination-Associated modules related to MM via WGCNA

**Fig. 1:**
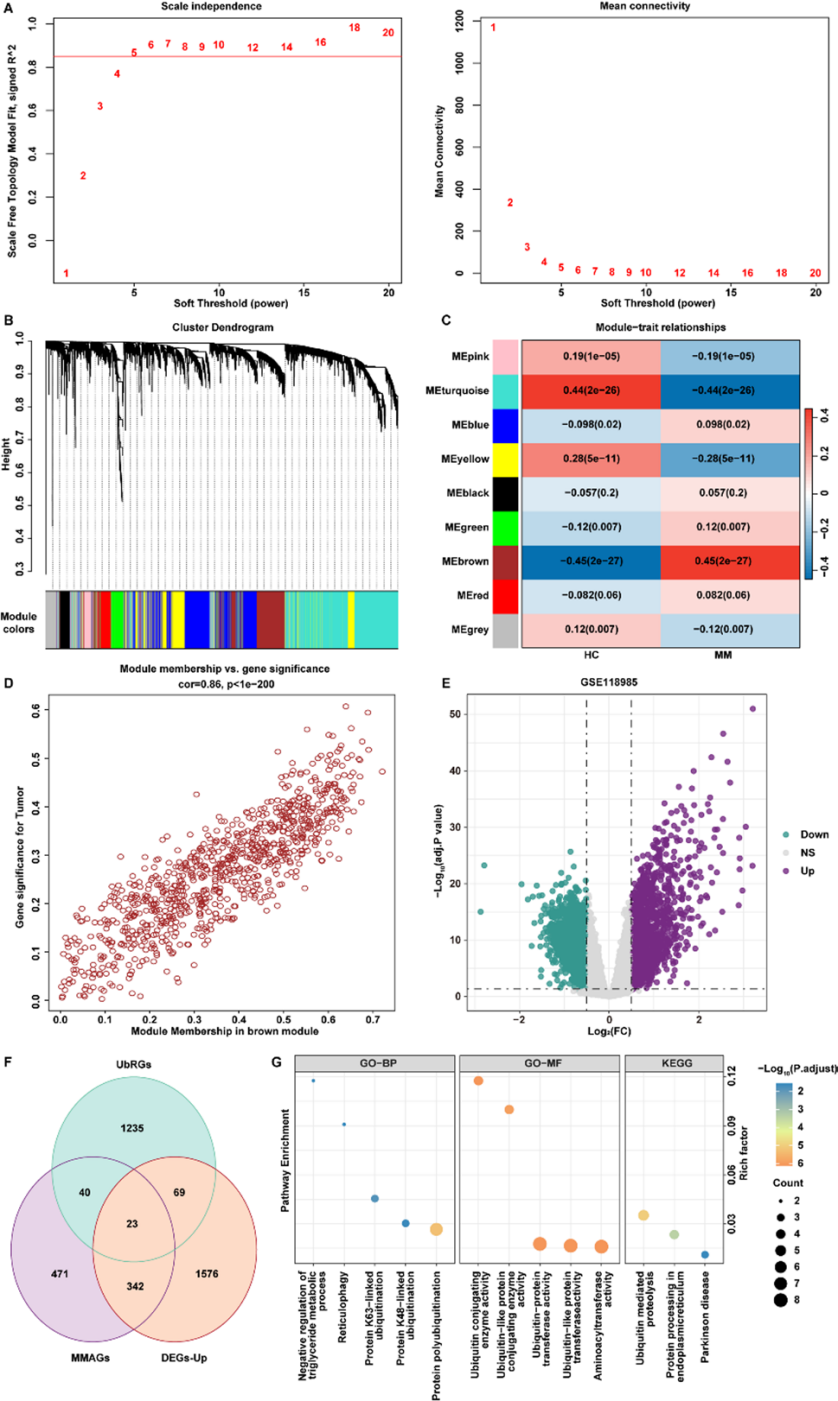
Identification and Functional Annotation of Ubiquitination-Related Key Genes in MM. **A** Scale-free topology fit index and mean connectivity analysis under different soft-threshold powers. **B** Gene clustering dendrogram and identification of co-expression modules. **C** Identification of modules highly correlated with MM and control groups. **D** Scatter plot of genes in the brown module. **E** Volcano plot of DEGs between MM and healthy samples in the GSE118985 dataset. **F** Venn diagram showing the intersection of MMAGs, DEGs, and UbRGs. **G** Bubble plot of GO and KEGG pathway enrichment analyses for key ubiquitination-related DEGs

To identify co-expression modules closely associated with MM, we constructed a weighted gene co-expression network using transcriptomic data from the GSE118985 dataset via the WGCNA algorithm. By evaluating the relationship between the soft-thresholding power (β), scale-free topology fit index (R²), and mean connectivity, a soft-threshold of β = 5 was selected as the optimal parameter for network construction (Fig. 1A). Hierarchical clustering of genes with high expression levels yielded nine distinct co-expression modules (Fig. 1B). Correlation analysis between module eigengenes and MM traits revealed a significant positive association between the brown module and the MM phenotype (*r* = 0.45, *p* = 2e − 27) (Fig. 1C). A total of 876 genes from the brown module, which showed a strong correlation with MM, were subsequently selected for further analysis and designated as MM-associated genes (MMAGs) (Fig. 1D, Table S2).

Further differential expression analysis between MM patients and healthy controls (HC) in the GSE118985 dataset identified 3,515 DEGs based on the criteria of |log₂FC| > 0.5 and FDR < 0.05. Of these, 2,010 genes were significantly upregulated and 1,505 were downregulated in the MM group compared to HC (Fig. 1E, Table S3). In this research, we focus on the 2010 upregulated DEGs for further analysis. To refine the gene set, we intersected the 2,010 upregulated DEGs, the 876 MMAGs identified by WGCNA, and the 1,367 UbRGs (Fig. 1F). This integrative analysis yielded 23 ubiquitination-related DEGs specifically associated with MM (Fig. 1F, Table S3).

Subsequently, functional enrichment analysis was performed on the 23 identified ubiquitination-related DEGs. GO analysis revealed significant enrichment across 32 GO terms, including 19 BP categories such as protein polyubiquitination, protein K63-linked ubiquitination, and negative regulation of triglyceride metabolic process, and 13 MF terms including ubiquitin-protein transferase activity, ubiquitin-like protein transferase activity, and aminoacyl transferase activity (Fig. 1G, Table S4). KEGG pathway analysis further indicated that these genes were predominantly enriched in three critical pathways: ubiquitin-mediated proteolysis, protein processing in the endoplasmic reticulum, and Parkinson disease (Fig. 1G, Table S4).

### Construction of the PPI Network and Identification of the Key Ubiquitination Gene Ring Finger Protein 25 (RNF25)

**Fig. 2:**
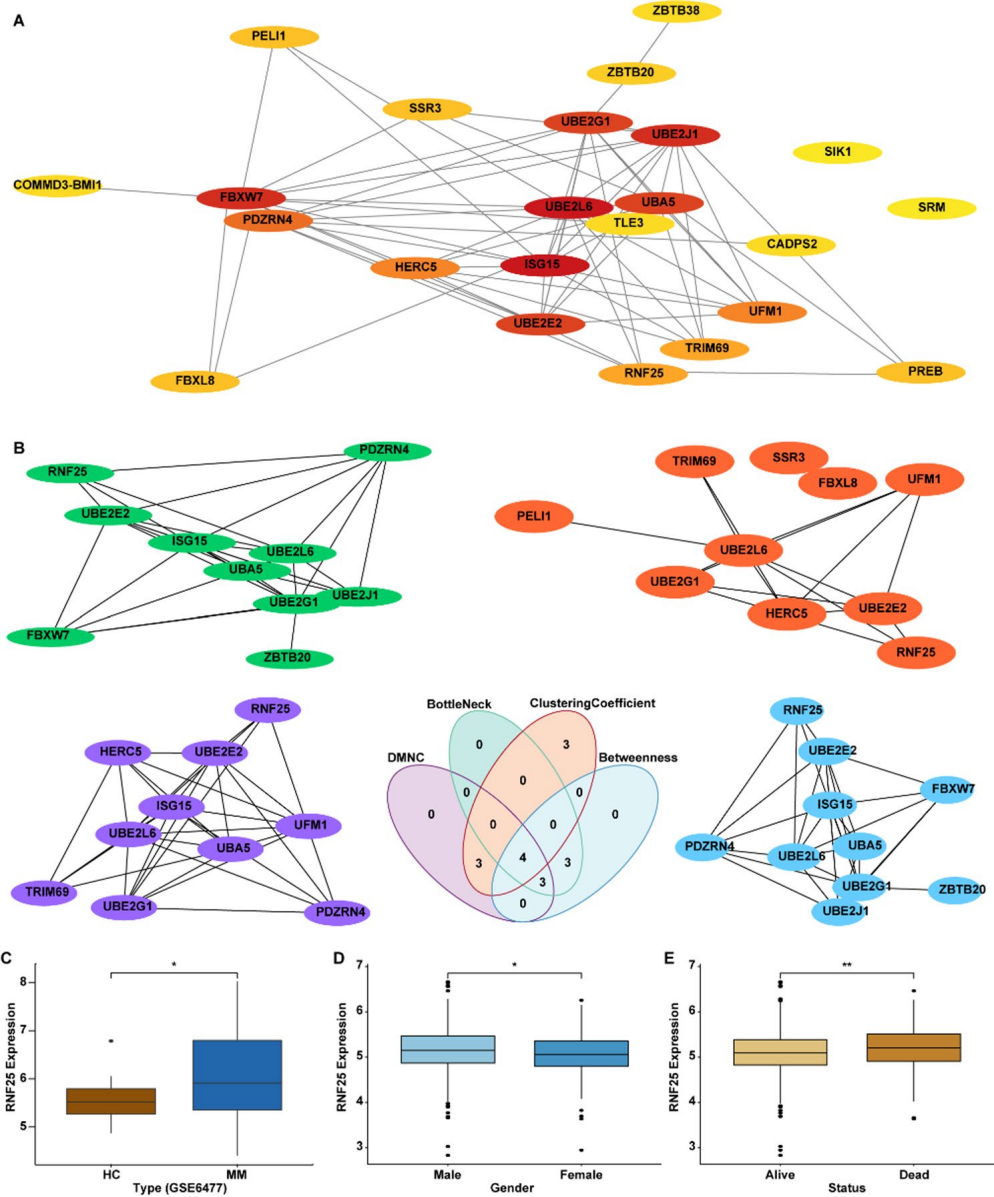
Identification and Expression Profiling of the Key Ubiquitination-Related Gene RNF25 in MM. **A** PPI network construction of MM-associated UbRGs. **B** Identification of hub genes using four algorithms: DMNC, BottleNeck, Betweenness, and Clustering Coefficient. **C** Expression differences of RNF25 between MM patients and HC in GSE6477 dataset. **D**–**E** RNF25 expression analysis across gender (**D**) and survival status (**E**) subgroups based on the MMRF-CoMMpass dataset. *P < 0.05, **P < 0.01

To further investigate the interactions among the 23 differentially expressed UbRGs associated with MM, a PPI network was constructed using the STRING online database. The resulting network consisted of 23 nodes and 63 edges, with an average node degree of 6 (Fig. 2A).

To identify hub genes within this network, we applied four topological algorithms: DMNC, Bottleneck, Betweenness Centrality, and Clustering Coefficient. Cross-comparison of the results revealed four genes consistently ranked as network hubs across all methods—UBE2G1, UBE2L6, UBE2E2, and RNF25 (Fig. 2B). Among these, RNF25 has been reported to be involved in various cancers [21–23]; however, its role in MM remains unexplored. Therefore, RNF25 was selected for further investigation in this study. Subsequent expression analysis of the GSE6477 dataset demonstrated that RNF25 was significantly upregulated in MM samples compared to HC (Fig. 2C). Moreover, analysis of the MMRF-CoMMpass cohort revealed that RNF25 expression was higher in males than in females, and was elevated in deceased patients compared with survivors, suggesting its potential association with adverse clinical outcomes (Figs. 2D-E).

**Fig. 3:**
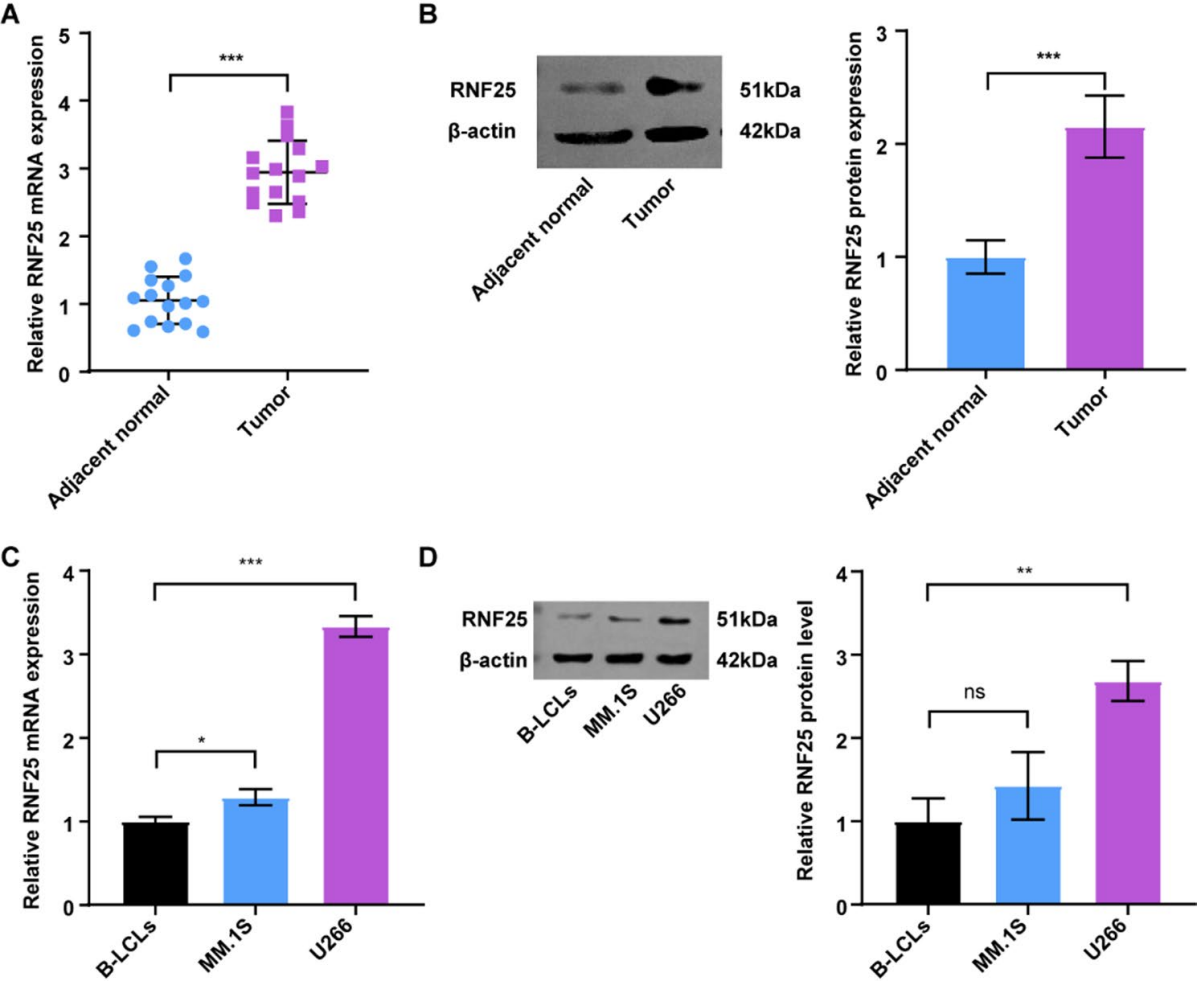
RNF25 is highly expressed in MM tissues and cell lines. **A** Relative RNF25 mRNA expression in MM tumor tissues and paired adjacent normal tissues. **B** Western blot and quantification showing RNF25 protein levels in MM tumor tissues versus adjacent normal tissues. **C** qRT-PCR analysis of RNF25 mRNA expression in B-LCLs, MM.1 S, and U266 cells. **D** Western blot and quantification of RNF25 protein expression in B-LCLs, MM.1 S, and U266 cells. *n* = 15 (qRT-PCR), Data are presented as mean ± SD. ***p < 0.001.

Building on these observations, we next sought to validate RNF25 expression in clinical and cellular samples. In paired tumor and adjacent normal tissues, both qRT-PCR and western blot analyses revealed that RNF25 was significantly upregulated in MM tissues relative to adjacent normal tissues (Figs. 3A–B). Consistently, in cellular models, we examined RNF25 expression in B-LCLs, MM.1 S, and U266 cells. qRT-PCR and western blot results confirmed that RNF25 was markedly overexpressed in MM cells, with the highest expression observed in U266 cells (Figs. 3C–D).

### RNF25 exhibits promising diagnostic potential and is significantly associated with poor prognosis in MM patients

**Fig. 4:**
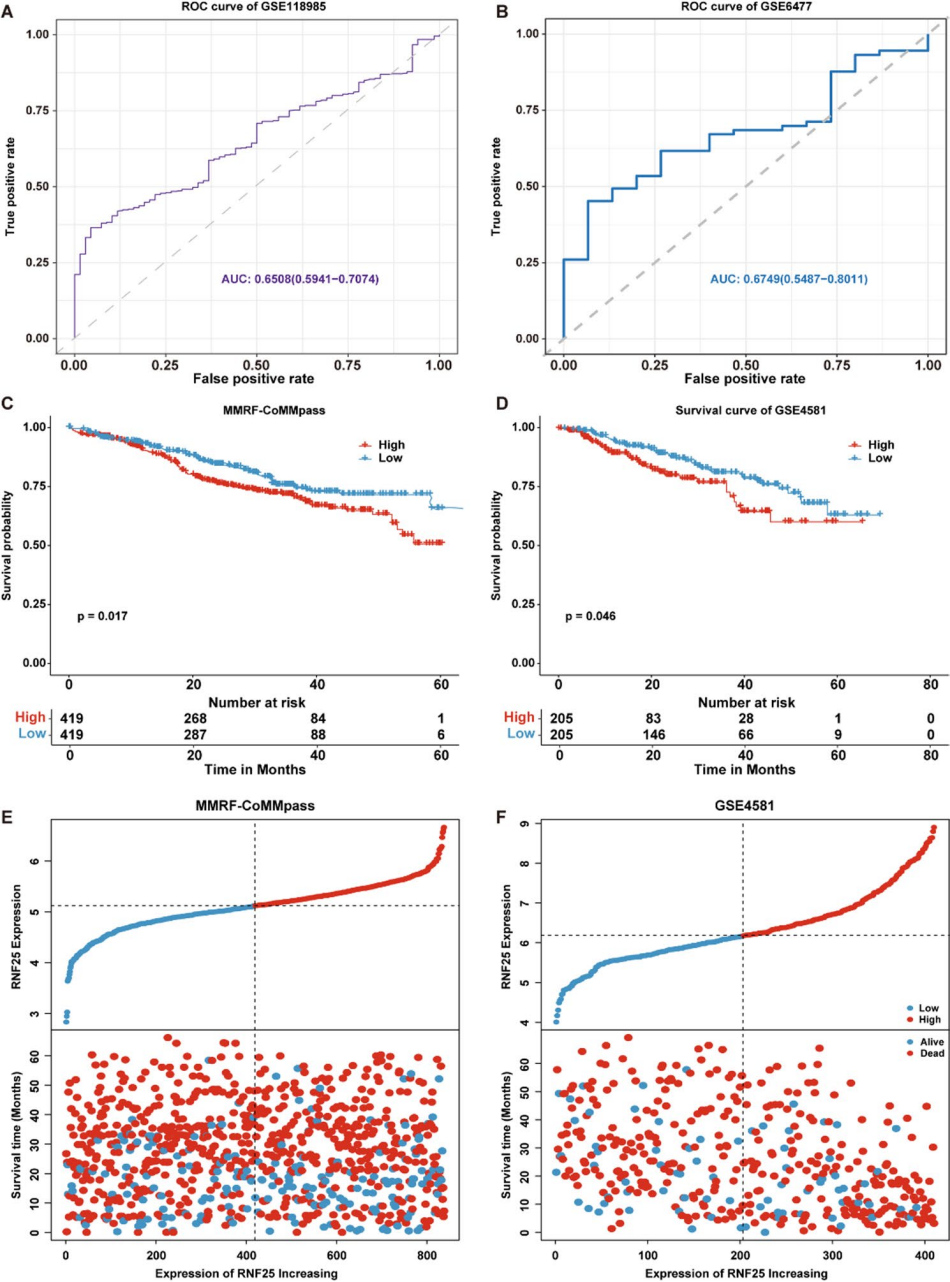
Diagnostic and prognostic relevance of RNF25 in MM. **A**–**B** ROC curve analysis of RNF25 in the GSE118985 (**A**) and GSE6477 (**B**) datasets. **C**–**D** Kaplan–Meier survival analysis of OS based on RNF25 expression in the MMRF-CoMMpass (**C**) and GSE4581 (**D**) cohorts. **E**–**F** Distribution plots of RNF25 expression with MMRF-CoMMpass (**E**) and GSE4581 (**F**)

To evaluate the diagnostic capability of RNF25 in MM, ROC curve analyses were performed in two independent datasets (GSE118985 and GSE6477) to assess its diagnostic performance in distinguishing MM patients from healthy controls. The results demonstrated moderate diagnostic efficacy, with AUC values of 0.650 in the GSE118985 dataset (Fig. 4A) and 0.6749 in the GSE6477 dataset (Fig. 4B), suggesting RNF25 may serve as a potential diagnostic biomarker for MM. Next, Kaplan–Meier survival analyses were conducted in the MMRF-CoMMpass and GSE4581 cohorts to assess the prognostic value of RNF25 in MM. In both datasets, patients with high RNF25 expression exhibited significantly shorter overall survival (OS) compared to those with low expression (MMRF-CoMMpass: *p* = 0.017; GSE4581: *p* = 0.046; Figs. 4C-D). Survival distribution plots further demonstrated that higher RNF25 expression was associated with shorter survival duration, and the proportion of deceased patients was markedly elevated in the high-expression group (Figs. 4E-F).

**Fig. 5:**
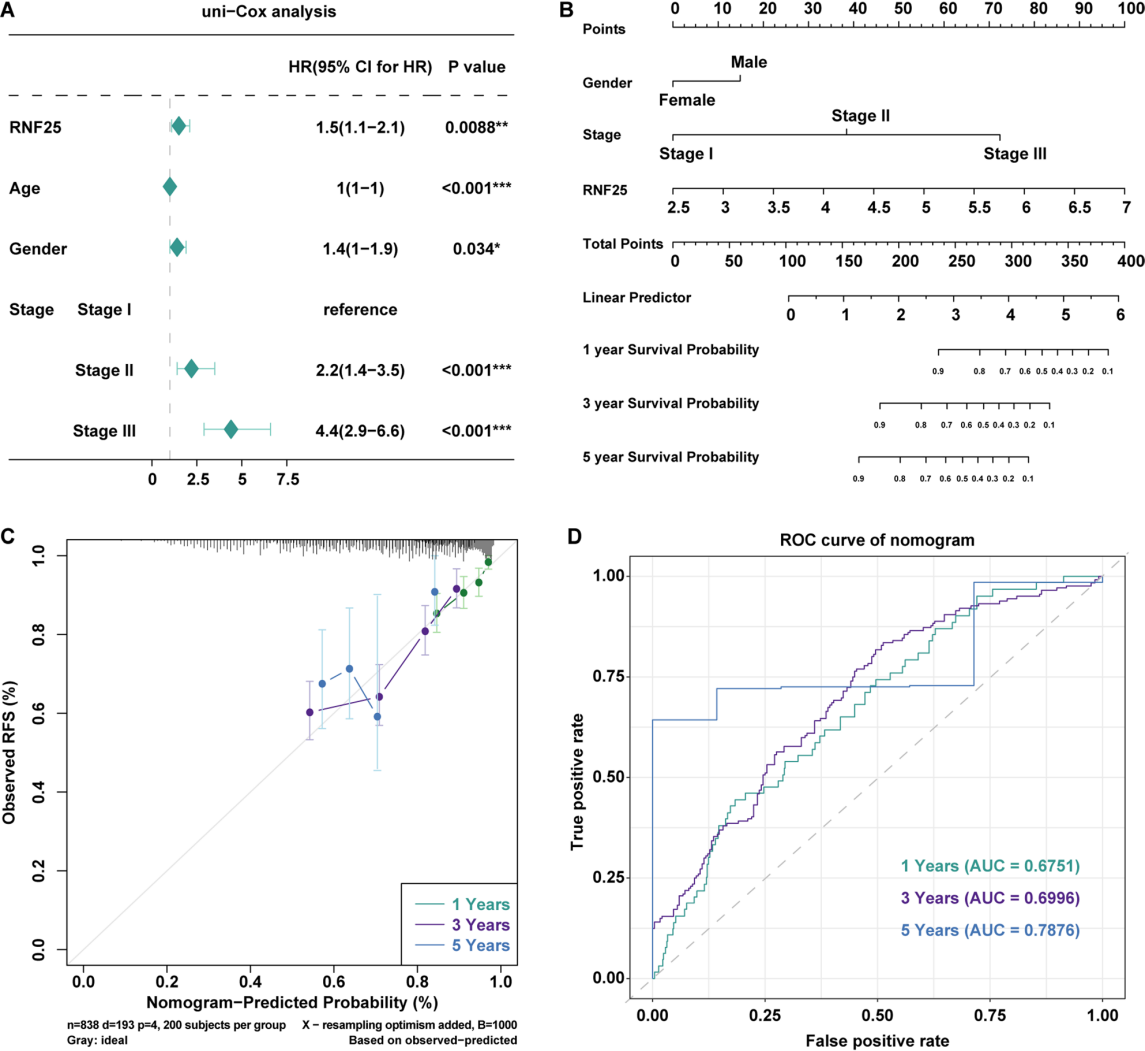
Construction and prognostic evaluation of a multivariate nomogram model based on RNF25 expression. **A** Univariate Cox regression analysis of RNF25 and clinical variables. **B** Nomogram integrating RNF25, gender, and clinical stage. **C** Calibration curves for 1-, 3-, and 5-year survival probability. **D** Time-dependent ROC curves for the nomogram model

To evaluate the independent prognostic value of RNF25, we summarized the clinical characteristics of the patients, including age, sex, and disease stage (Table S5), and performed univariate Cox regression analyses using the MMRF-CoMMpass dataset. The results indicated that high RNF25 expression was significantly associated with poor prognosis in MM patients (HR = 1.5, 95% CI: 1.1–2.1, *p* = 0.0088). Additionally, age (*p* < 0.001), sex (*p* = 0.034), and pathological stage (stage II and III, both *p* < 0.001) were also significantly associated with prognosis (Fig. 5A). Subsequently, a multivariate Cox regression model was constructed by integrating RNF25 expression, sex, and clinical stage. A nomogram was generated to predict 1-, 3-, and 5-year OS probabilities in MM patients (Fig. 5B). The model showed that patients with higher RNF25 expression had worse predicted outcomes. Calibration curves demonstrated good agreement between predicted and observed survival at 1, 3, and 5 years (Fig. 5C), indicating robust model performance. Time-dependent ROC analysis further validated the prognostic accuracy of the nomogram, with AUC values of 0.637 (1-year), 0.696 (3-year), and 0.798 (5-year), respectively (Fig. 5D). These findings suggest that RNF25, in combination with clinical parameters, may serve as a reliable independent prognostic indicator for individualized prognosis assessment in MM.

### Enrichment pathway analysis

In the MMRF-CoMMpass dataset, patients were stratified into RNF25 high- and low-expression groups based on the median expression level (log₂[TPM] = 5.12), and differential expression analysis was performed between the two groups. The resulting DEGs were subsequently subjected to GO, KEGG, and GSEA.

**Fig. 6:**
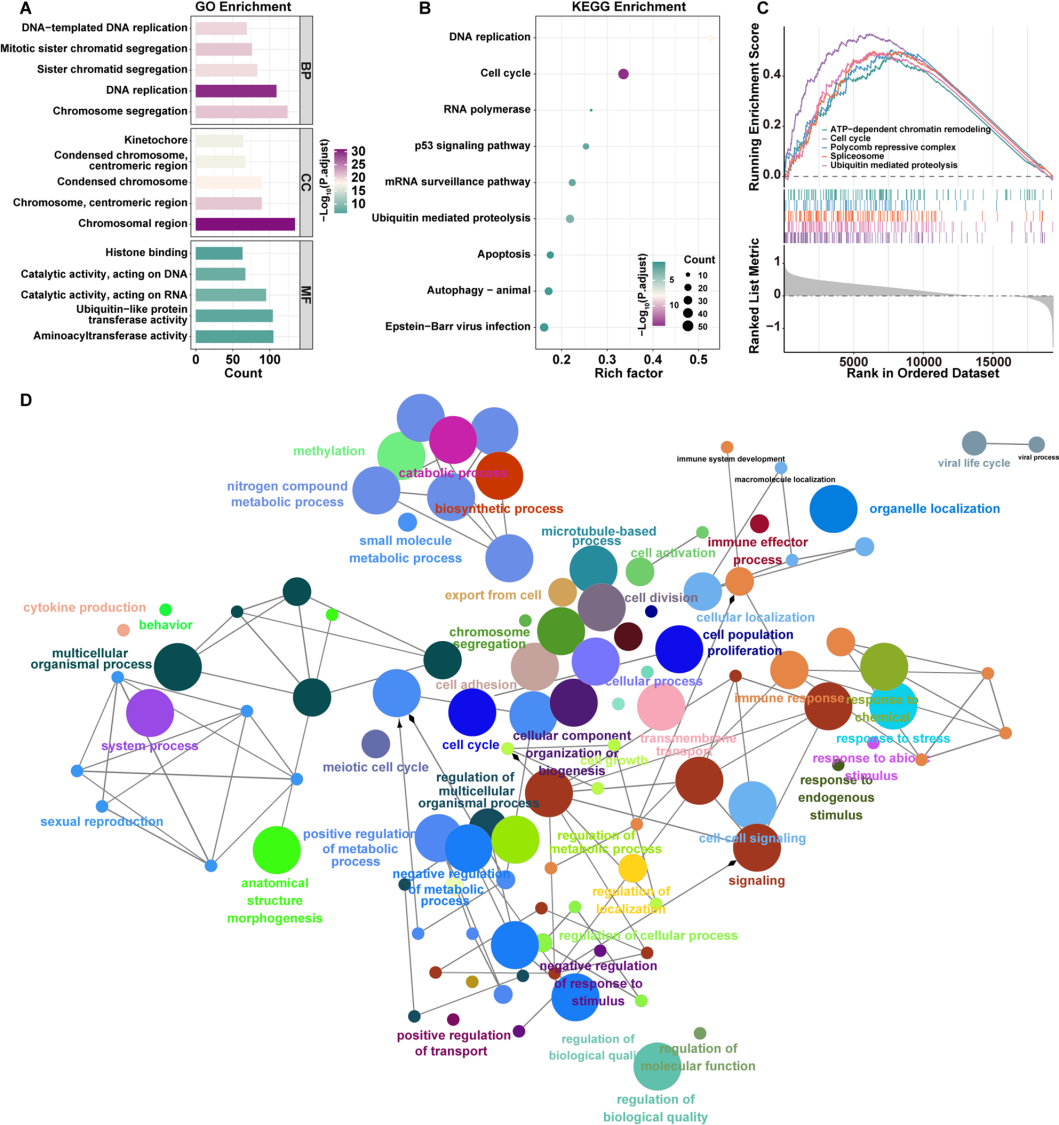
Functional enrichment and network visualization analysis of RNF25-associated DEGs. **A** GO enrichment analysis results. **B** KEGG pathway enrichment bubble plot. **C** GSEA enrichment analysis visualization. **D** GO enrichment network constructed using Metascape

GO enrichment analysis revealed that these DEGs were significantly enriched in 379 BP terms, primarily related to cell cycle regulation, including DNA-templated DNA replication, mitotic sister chromatid segregation, and sister chromatid segregation. For CC, 135 enriched terms were identified, mainly associated with chromosomal structures such as chromosomal region, chromosome, centromeric region, and condensed chromosome. In terms of MF, 69 significantly enriched terms were observed, including aminoacyltransferase activity, ubiquitin-like protein transferase activity, and catalytic activity acting on RNA (Fig. 6A, Table S6). KEGG pathway analysis further demonstrated that these DEGs were significantly enriched in 27 pathways, notably including cell cycle, DNA replication, and the p53 signaling pathway, all of which are classical cancer-related pathways (Fig. 6B, Table S6).

Complementary GSEA confirmed that the RNF25 high-expression group was significantly enriched for hallmark pathways such as cell cycle, ATP-dependent chromatin remodeling, Polycomb repressive complex, spliceosome, and ubiquitin-mediated proteolysis (Fig. 6C, Table S6).

Furthermore, a functional annotation network based on GO terms clustered these biological processes into distinct functional modules. Collectively, RNF25-associated genes were markedly enriched in tightly interconnected biological processes, including cell cycle regulation, immune response, and transmembrane transport (Fig. 6D, Table S7), highlighting the potential multifaceted role of RNF25 in MM pathogenesis.

### RNF25 expression is significantly associated with immune cell infiltration and immune function in MM

**Fig. 7:**
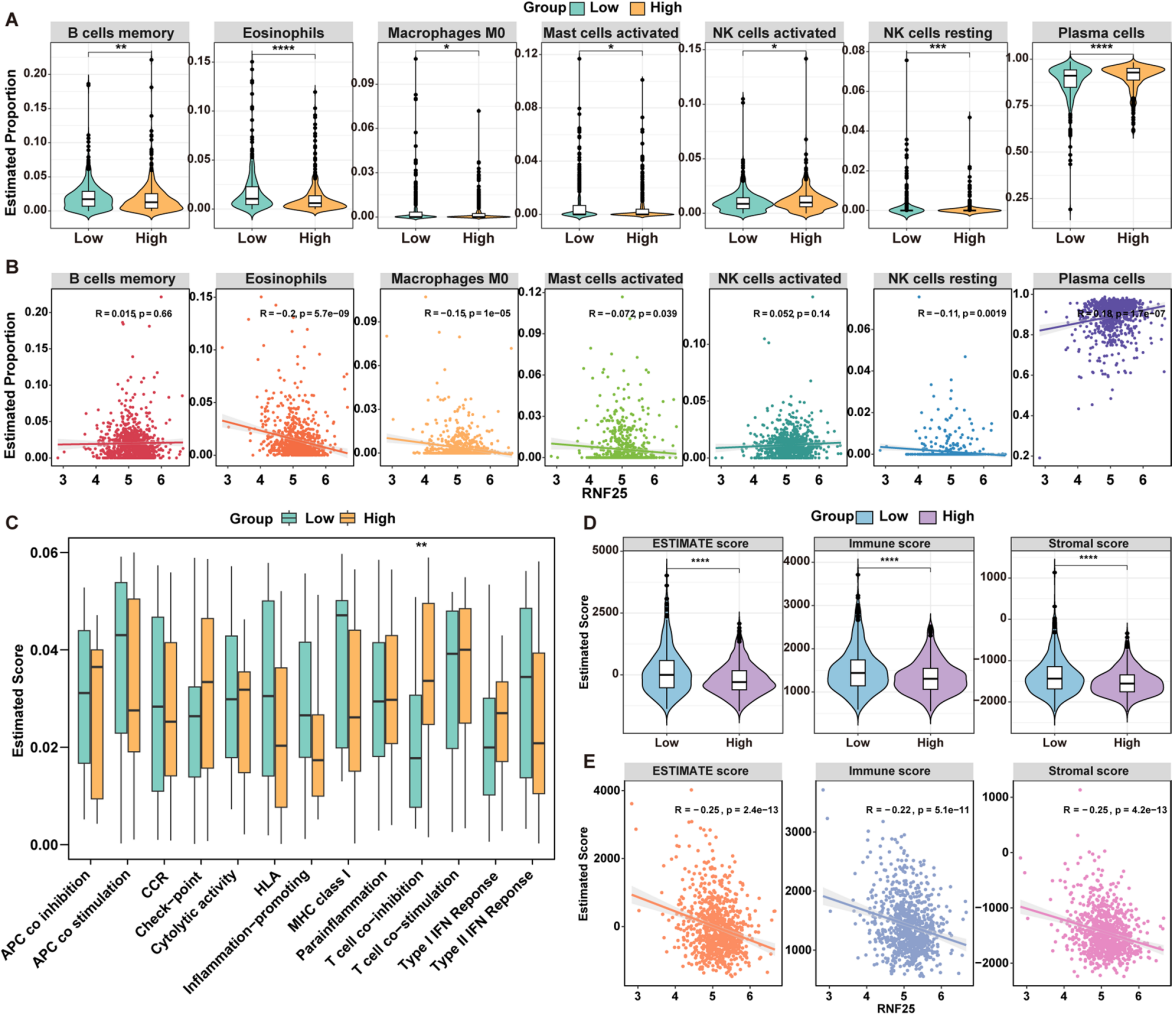
Association between RNF25 expression and immune microenvironment in MM. **A** CIBERSORT algorithm was used to evaluate differences in the infiltration levels of immune cell subtypes between RNF25 high- and low-expression groups. **B** Correlation analysis between RNF25 expression and significantly altered immune cell subtypes. **C** ssGSEA analysis of 13 immune-related functional pathways between RNF25 high- and low-expression groups. **D** Comparison of ESTIMATE Score, Immune Score, and Stromal Score between RNF25 high- and low-expression groups. **E** Correlation of RNF25 expression with ESTIMATE Score, Immune Score, and Stromal Score. *P < 0.05, **P < 0.01, ***P < 0.001, ****P < 0.0001

To explore the potential role of RNF25 in the immune microenvironment of MM, immune cell infiltration analysis was performed using the MMRF-CoMMpass dataset. Patients were stratified into high- and low-RNF25 expression groups, and immune cell proportions were estimated using the CIBERSORT algorithm for 22 subtypes. A total of 7 immune cell subpopulations exhibited significant differences in infiltration levels between the two groups, including memory B cells, eosinophils, M0 macrophages, activated mast cells, activated NK cells, resting NK cells, and plasma cells (Fig. 7A). Correlation analysis was subsequently conducted to assess the relationship between RNF25 expression and immune cell infiltration levels. RNF25 expression was found to be positively correlated with plasma cell infiltration (*R* = 0.18, *p* = 1.7e − 07), whereas negative correlations were observed with eosinophils (*R* = − 0.20, *p* = 5.7e − 09), M0 macrophages (*R* = − 0.15, *p* = 1e − 05), resting NK cells (*R* = − 0.11, *p* = 0.0019), and activated mast cells (*R* = − 0.072, *p* = 0.039) (Fig. 7B). Although memory B cells and activated NK cells showed differences in infiltration levels between groups, their correlation with RNF25 expression was not statistically significant (*p* > 0.05).

To evaluate the effect of RNF25 on immune function, we employed single-sample Gene Set Enrichment Analysis (ssGSEA) to score 13 immune-related functional pathways. Notably, the T cell co-inhibition pathway was significantly upregulated in the RNF25 high-expression group (*p* < 0.001) (Fig. 7C), suggesting that RNF25 may contribute to immune evasion in MM by enhancing T cell immunosuppressive activity. Furthermore, the ESTIMATE algorithm was used to assess the overall immune and stromal components of the tumor microenvironment. The results revealed that the ESTIMATE Score, Immune Score, and Stromal Score were all significantly lower in the RNF25 high-expression group (all *p* < 0.001), and each of these scores exhibited a significant negative correlation with RNF25 expression (Figs. 7D–E): ESTIMATE Score: *R* = − 0.25, *p* = 2.4e − 13; Immune Score: *R* = − 0.22, *p* = 5.1e − 11; Stromal Score: *R* = − 0.25, *p* = 4.2e − 13.

### RNF25 is associated with drug sensitivity and serves as a potential binding target for Temozolomide

**Fig. 8:**
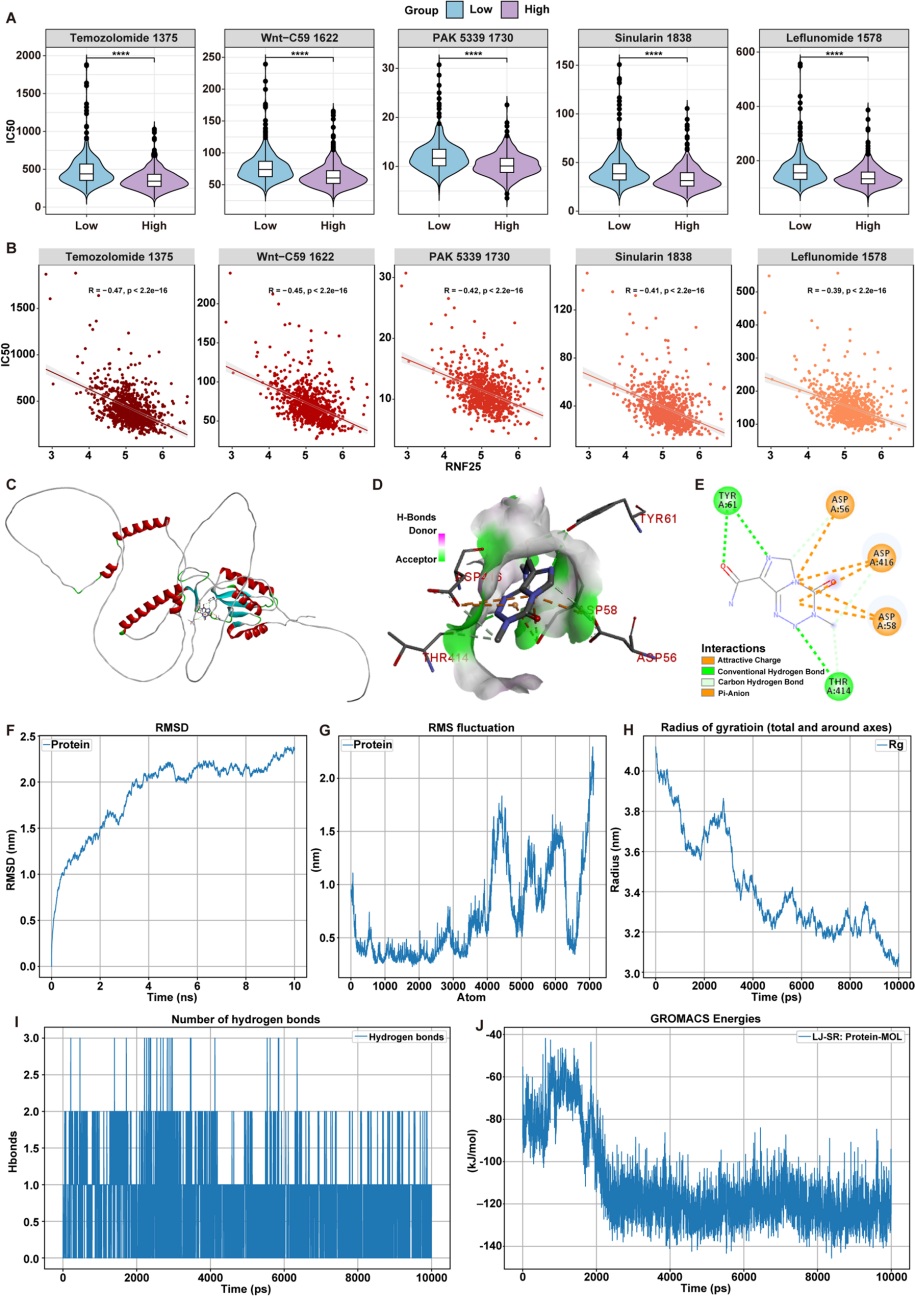
Association Between RNF25 Expression and Drug Sensitivity, and Molecular Dynamics Analysis. **A** IC_50_ comparisons of five anticancer drugs between high and low RNF25 expression groups. **B** Spearman correlation analysis between RNF25 expression and IC_50_ values of the five drugs. **C**–**E** Molecular docking analysis of Temozolomide and RNF25, including protein structure overview (**C**), binding pocket visualization (**D**), and 2D interaction map of key amino acid residues (**E**). **F**–**J** Molecular dynamics simulation results of the Temozolomide–RNF25 complex, including RMSD (**F**), RMSF (**G**), Rg (**H**), number of hydrogen bonds (**I**), and LJ-SR interaction energy (**J**). ****P < 0.0001

To evaluate the potential role of RNF25 in drug sensitivity prediction, the MMRF-CoMMpass dataset was analyzed using the oncoPredict tool to estimate the IC_50_ for 198 anticancer drugs across all samples. Correlation analysis between RNF25 expression and drug response revealed that the IC_50_ values of Temozolomide, Wnt-C59, PAK, Sinularin, and Leflunomide were significantly lower in the RNF25 high-expression group (****P < 0.0001) (Fig. 8A). Spearman correlation analysis further confirmed significant negative correlations between RNF25 expression and the IC_50_ values of all five drugs (Fig. 8B), suggesting that patients with elevated RNF25 expression may be more sensitive to these agents.

Among them, Temozolomide—a classical alkylating agent widely used in the standard treatment of glioblastoma [24]—has not yet been extensively studied in MM. Given its clinical relevance and potential, Temozolomide was selected as the representative candidate for subsequent mechanistic investigation. To explore the binding capacity and potential molecular interaction between Temozolomide and RNF25, molecular docking analysis was conducted. The results demonstrated that Temozolomide stably fits into the active binding pocket of the RNF25 protein (Fig. 8C), forming stable interactions with key amino acid residues including ASP56, ASP58, ASP416, TYR61, and THR414 (Fig. 8D). Two-dimensional interaction analysis further revealed that Temozolomide forms multiple stabilizing interactions—such as conventional hydrogen bonds, attractive charge interactions, and π-anion interactions—with these residues (Fig. 8E), indicating a robust binding affinity.

To assess the dynamic stability of the Temozolomide–RNF25 complex, molecular dynamics simulations were performed. RMSD analysis showed that the complex rapidly achieved a stable conformation within the first 2 ns and maintained consistent structural fluctuations within the 2.0–2.3 nm range throughout the simulation (Fig. 8F). RMSF analysis indicated low flexibility across most protein regions, with only the N- and C-terminal tails and several flexible loops exhibiting minor fluctuations, while the core binding region remained stable (Fig. 8G). The Rg gradually decreased and stabilized, suggesting the complex transitioned from a loose to a compact state (Fig. 8H). Hydrogen bond analysis indicated the formation of 1–2 stable hydrogen bonds between Temozolomide and RNF25 throughout the entire simulation period (Fig. 8I), supporting the persistence of the interaction. In addition, Lennard-Jones short-range (LJ-SR) interaction energy analysis revealed a rapid decline in potential energy at the start of the simulation, which then stabilized around − 120 to − 130 kJ/mol without major fluctuations (Fig. 8J), further indicating strong and stable non-bonded interactions between the two molecules.

### Knockdown of RNF25 affects the cellular functions of MM cells

**Fig. 9:**
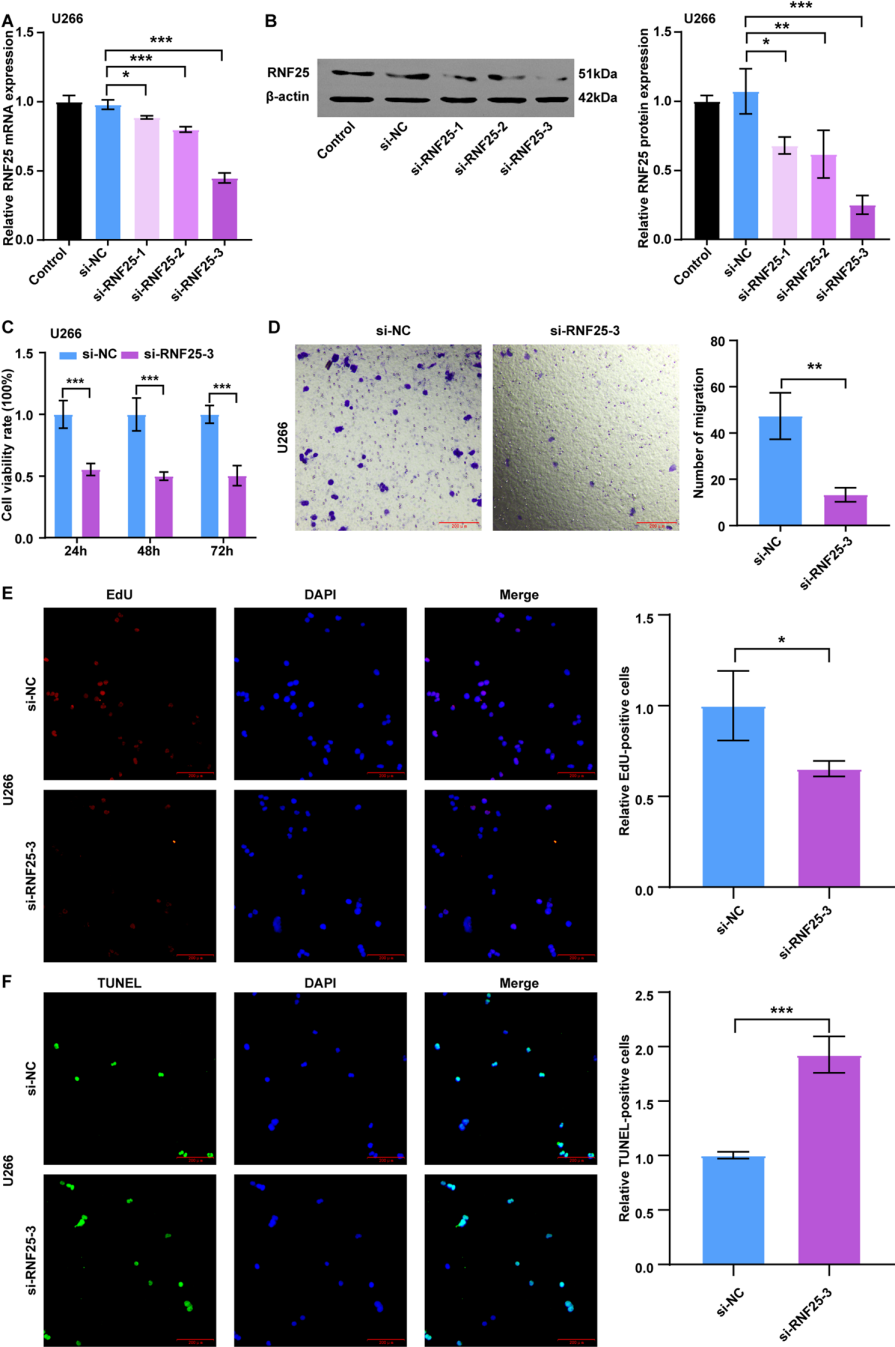
Functional assays following RNF25 knockdown in U266 cells. **A** qRT-PCR analysis of RNF25 mRNA expression in U266 cells treated with control, si-RNF25-1, si-RNF25-2, or si-RNF25-3. **B** Western blot analysis of RNF25 protein expression and corresponding quantification in U266 cells treated with control, si-RNF25-1, si-RNF25-2, or si-RNF25-3. **C** CCK-8 assay evaluating the effect of si-RNF25-3 treatment on U266 cell viability. **D** Transwell migration assay assessing the migratory ability of U266 cells after si-RNF25-3 treatment. **E** EdU incorporation assay detecting DNA synthesis and proliferative activity in U266 cells following si-RNF25-3 treatment. **F** TUNEL assay detecting apoptosis in U266 cells following transfection with si-RNF25-3. Data are presented as mean ± SD. *P < 0.05, **P < 0.01, *P < 0.001

To further investigate the functional role of RNF25 in MM, three siRNAs (si-RNF25-1, si-RNF25-2, si-RNF25-3) were designed to silence its expression in two MM cell lines, U266 and MM.1 S. As shown in Figs. 9A-B and Figs. S1A-B, treatment with either a single siRNA or a siRNA pool significantly downregulated RNF25 expression in both cell lines. Among the three siRNAs, si-RNF25-3 demonstrated the highest knockdown efficiency and was used in subsequent experiments (Figs. 9A-B, Figs. S1A-B). CCK-8 assay results revealed that si-RNF25-3 notably decreased cell viability in both U266 and MM.1 S cells (Fig. 9C, Figs. S1C-D). Importantly, RNF25 overexpression significantly reversed the reduction in cell viability induced by RNF25 knockdown in U266 cells (Fig. S2A-C).

Furthermore, si-RNF25-3 significantly inhibited the migration and proliferation in U266 cells (Figs. 9D-E), while markedly promoting apoptosis (Fig. 9F). Collectively, these findings indicate that RNF25 may exert an oncogenic role in MM.

## Discussion

RNF25 is an E3 ubiquitin ligase containing a RING domain and is a member of the RING finger protein family, playing essential roles in various physiological and pathological processes [21]. Previous studies have shown that in non-small cell lung cancer, RNF25 can form a complex with the NF-κB subunit RelA/p65 and promote its ubiquitination, thereby enhancing NF-κB signaling and participating in inflammation, apoptosis, and immune regulation [23]. In cancer contexts, RNF25 has also been reported to exhibit oncogenic activity. For instance, in hepatocellular carcinoma, RNF25 mediates the degradation of E-cadherin under oxidative stress conditions, thereby promoting tumor cell migration and metastasis [22]; in colorectal cancer, RNF25 cooperates with CARM1 to ubiquitinate and degrade ACSL4, suppressing ferroptosis and enhancing tumor cell survival [25]. However, the functional significance of RNF25 in MM remains largely unexplored. Therefore, this study focused on elucidating the biological role of RNF25 in MM.

This study systematically integrated multiple public transcriptomic datasets of MM and identified RNF25 as a UPS-related gene closely associated with MM. RNF25 was highly expressed in MM tissues across datasets and further validated in clinical samples. Elevated RNF25 expression was associated with poor overall survival and identified as an independent adverse prognostic factor in MM. Functional analyses demonstrated that RNF25 knockdown in U266 cells reduced cell viability, proliferation, and migration, while promoting apoptosis.

RING finger proteins, as a major class of E3 ubiquitin ligases, play an important role in the ubiquitination of core histones involved in chromatin remodeling [26]. Chromatin remodeling plays a critical role in maintaining genome integrity and regulating transcription, and its dysregulation has been implicated in impaired DNA repair, replication stress, cellular senescence, metastasis, and immune evasion [27]. Recent sequencing studies in MM cohorts have shown that mutations in chromatin-regulatory genes are closely associated with progression-free survival, bone marrow plasma cell burden, and treatment response, indicating that chromatin remodeling is a key driver of clinical heterogeneity and may provide a potential basis for prognostic stratification [28]. Previous studies have shown that RNF20 promotes gene transcription and elongation by catalyzing histone H2B monoubiquitination, which in turn regulates chromatin remodeling complexes [29]. In addition, RNF20–RNF40–mediated histone ubiquitination facilitates the recruitment of transcription factors and RNA polymerase II while reshaping chromatin structure, thereby playing a key role in transcriptional regulation [30]. Taken together, these findings demonstrate that the RNF family is closely linked to chromatin remodeling. The GSEA results from this study revealed that samples with high RNF25 expression were significantly enriched in ATP-dependent chromatin remodeling pathways, suggesting a potential association between RNF25 and chromatin remodeling in MM.

In addition, GSEA analysis also revealed that RNF25 is closely associated with the cell cycle. Previous studies have shown that E3 ubiquitin ligases play an important role in regulating the cell cycle and thereby influence cancer progression [31]. For instance, RNF8 regulates mitosis and cytokinesis through its E3 ubiquitin ligase activity to maintain cell cycle stability, whereas RNF8 dysfunction leads to cytokinesis failure and cell cycle dysregulation, ultimately promoting tumor progression [32]. Based on these findings, we hypothesize that RNF25, as a RING-type E3 ubiquitin ligase, may promote the development of MM by regulating cell cycle progression, although the precise mechanisms remain to be further elucidated.

Furthermore, our GSEA analysis also found that high RNF25 expression was closely associated with ubiquitin-mediated proteolysis. Previous studies have shown that activation of the NF-κB pathway requires ubiquitination of the inhibitory protein IκB followed by proteasome-mediated degradation, thereby facilitating the release of p65 from the inhibitory complex and its nuclear translocation to initiate target gene transcription [33]. Notably, the study by Li et al. demonstrated that in malignant renal tumors, the E3 ubiquitin ligase RNF25 does not act directly on p65, but instead regulates NF-κB signaling activation by targeting TRIP4 as a direct ubiquitination substrate [34]. These findings suggest that RNF25 may participate in and influence NF-κB pathway activity in MM through ubiquitination regulation mediated by the RNF25–TRIP4 axis. Nevertheless, further studies are required to fully elucidate the underlying molecular mechanisms.

Immune microenvironment analysis revealed that plasma cell infiltration was significantly increased in the RNF25 high-expression group and positively correlated with RNF25 expression. MM is a plasma cell-derived malignancy [35], and malignant plasma cells together with stromal cells can secrete various inflammatory and immunosuppressive cytokines, such as IL-6, IL-10, and TGF-β, collectively shaping an “immunosuppressive cytokine milieu” that attenuates the activation and effector functions of T cells and NK cells [36–38]. Notably, IL-10 can promote the expansion of regulatory T cells (Tregs) and inhibit dendritic cell maturation, thereby attenuating anti-tumor immunity [39]. These findings suggest a potential association between high RNF25 expression and a microenvironment that may have immunosuppressive effects.

Using ssGSEA, we evaluated 13 immune-related functional pathways and found that the T cell co-inhibition pathway was activated in the RNF25 high-expression group. T cell co-inhibition is a fundamental mechanism for maintaining immune homeostasis and is primarily mediated by immune checkpoint molecules such as PD-1, CTLA-4, and TIGIT, which negatively regulate T cell activation and effector functions [40]. In the tumor microenvironment, cancer cells can upregulate inhibitory ligands such as PD-L1 and GAL9, which bind to co-inhibitory receptors on T cells and induce T cell exhaustion, thereby facilitating immune evasion [41]. Previous studies have shown that tumor-infiltrating T cells often co-express multiple inhibitory receptors, a phenomenon known as “co-inhibitory co-expression,” which markedly impairs their cytotoxic capacity and antitumor responses [42]. Taken together with the activation of the T cell co-inhibition pathway, these findings suggest that high RNF25 expression may contribute to T cell dysfunction, thereby facilitating immune evasion and accelerating the progression of MM.

Finally, drug sensitivity predictions, combined with molecular docking analyses, indicated that RNF25-high samples may exhibit increased in silico sensitivity to several anticancer agents, including temozolomide, Wnt-C59, and PAK inhibitors, suggesting that RNF25 may serve as a predictive biomarker for therapeutic response. Temozolomide is an oral DNA-alkylating agent widely used in the treatment of glioblastoma [43]. Recently, research has shown that temozolomide has been studied in hematologic malignancies, such as primary central nervous system lymphoma and primary vitreoretinal lymphoma [44, 45]. In this study, we found that the predicted IC50 values were significantly negatively correlated with RNF25 expression, and computational modeling suggested a favorable binding mode between temozolomide and RNF25, collectively implying a potential interaction between temozolomide and RNF25. This suggests a possible role of temozolomide in MM, however, further studies are needed to confirm this potential.

This study has several limitations. First, it has been shown that CD138 is a common surface marker of plasma cells. By detecting CD138, it helps to identify and isolate malignant plasma cells, enabling more accurate diagnosis and treatment [46]. However, the transcriptome dataset analyzed in this study was derived from unsorted bone marrow samples, without prior enrichment of CD138⁺ plasma cells. Future studies can focus on the sorting of CD138⁺ plasma cells to further investigate the transcriptional programs associated with RNF25 in MM cells, in order to more accurately elucidate its role in the pathogenesis of MM. Second, our immune microenvironment analysis relied entirely on CIBERSORT-based deconvolution of bulk RNA-seq data and has not yet been supported by experimental approaches such as flow cytometry or single-cell sequencing. Therefore, the immune-related hypotheses proposed in this study will need to be further validated in future work. Third, the predicted interaction between temozolomide and RNF25 is based solely on molecular docking and molecular dynamics simulations. Such computational approaches cannot demonstrate true physical binding or functional inhibition, and future systematic biochemical assays and cell-based drug response experiments will be required to verify these in silico predictions.

## Conclusion

This study identified RNF25 as a key gene in MM. RNF25 was found to be highly expressed in MM tissues, where its high expression enhanced cells viability, proliferation, and migration while suppressing apoptosis. Moreover, high RNF25 expression was significantly associated with poor clinical outcomes and increased sensitivity to several anticancer drugs, including temozolomide. These findings highlight the clinical value of RNF25 as a potential prognostic biomarker and provide a theoretical basis for its development as a candidate therapeutic target in MM.

## Supplementary Information

Supplementary Material 1.

Supplementary Material 2.

Supplementary Material 3.

Supplementary Material 4.

Supplementary Material 5.

Supplementary Material 6.

Supplementary Material 7.

Supplementary Material 8.

Supplementary Material 9.

Supplementary Material 10.

## Data Availability

Data supporting the conclusions of this article are openly available in the GEO database at [https://www.ncbi.nlm.nih.gov/geo/](https:/www.ncbi.nlm.nih.gov/geo), reference numbers GSE118985, GSE6477 and GSE4581.
