# Supplementary figures and images for: RNF25 serves as a novel diagnostic and prognostic biomarker in multiple myeloma: a multi-cohort integrative analysis
Source: Hereditas. 2025 Dec 28;163:18. doi: 10.1186/s41065-025-00631-0 (PMC12853844; doi:10.1186/s41065-025-00631-0)

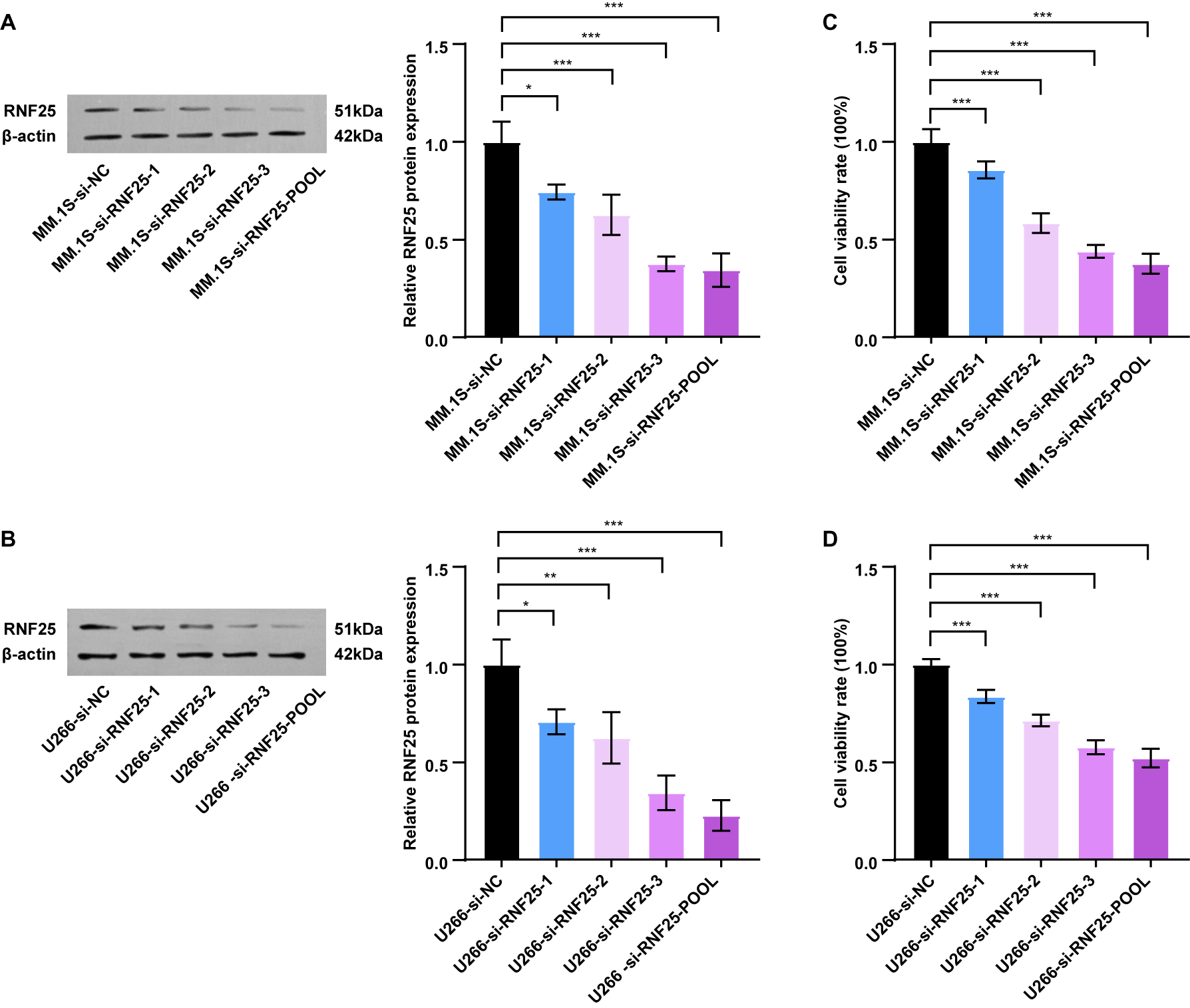

Supplement: Supplementary file 8 — Supplementary Material 8. [file 41065_2025_631_MOESM8_ESM.tif]

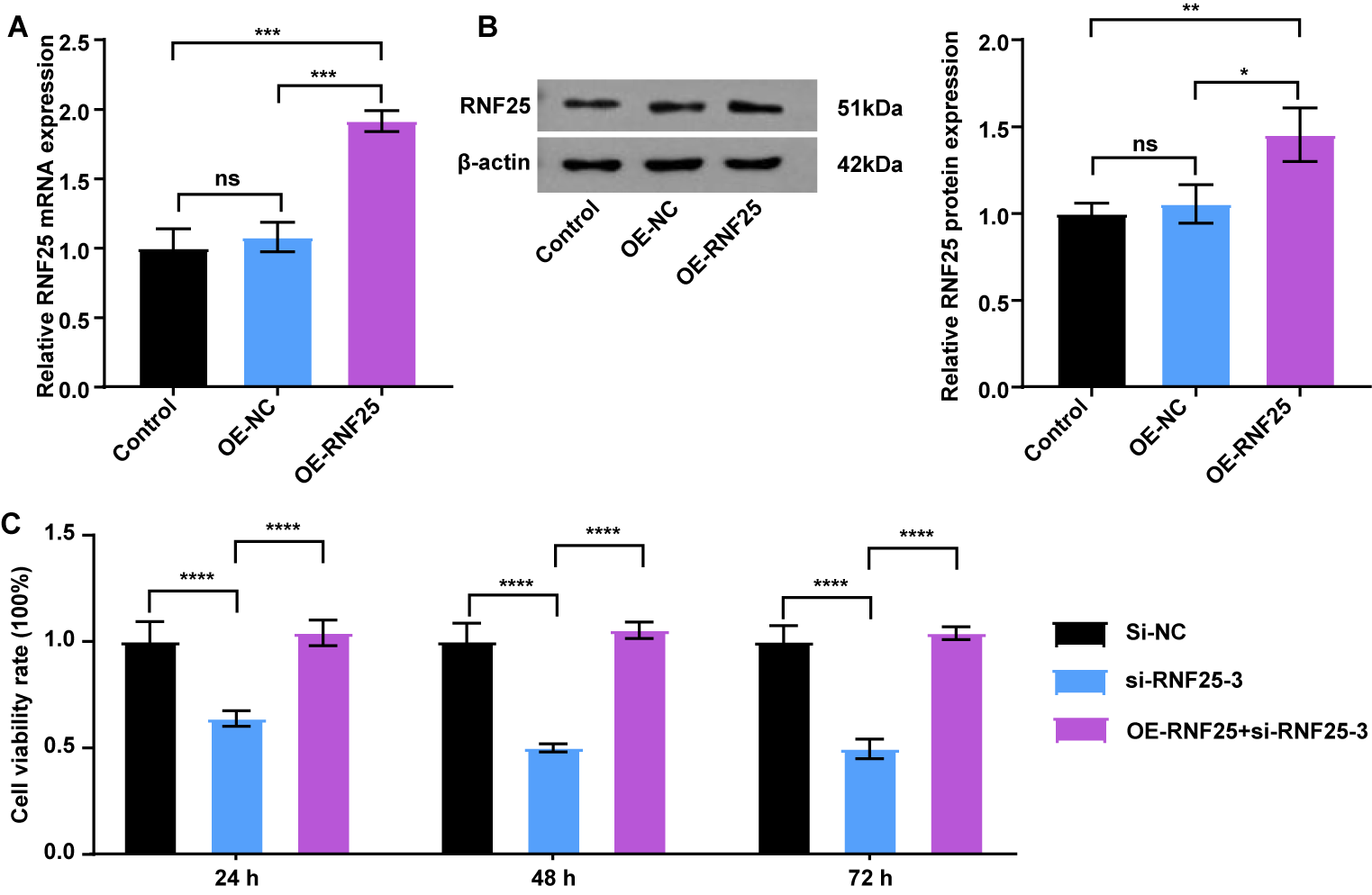

Supplement: Supplementary file 9 — Supplementary Material 9. [file 41065_2025_631_MOESM9_ESM.tif]
