# Supplementary material for: RNF25 serves as a novel diagnostic and prognostic biomarker in multiple myeloma: a multi-cohort integrative analysis
Source: Hereditas. 2025 Dec 28;163:18. doi: 10.1186/s41065-025-00631-0 (PMC12853844; doi:10.1186/s41065-025-00631-0)
Supplement: Supplementary file 10 — Supplementary Material 10. [file 41065_2025_631_MOESM10_ESM.pdf]

Fig. 3B

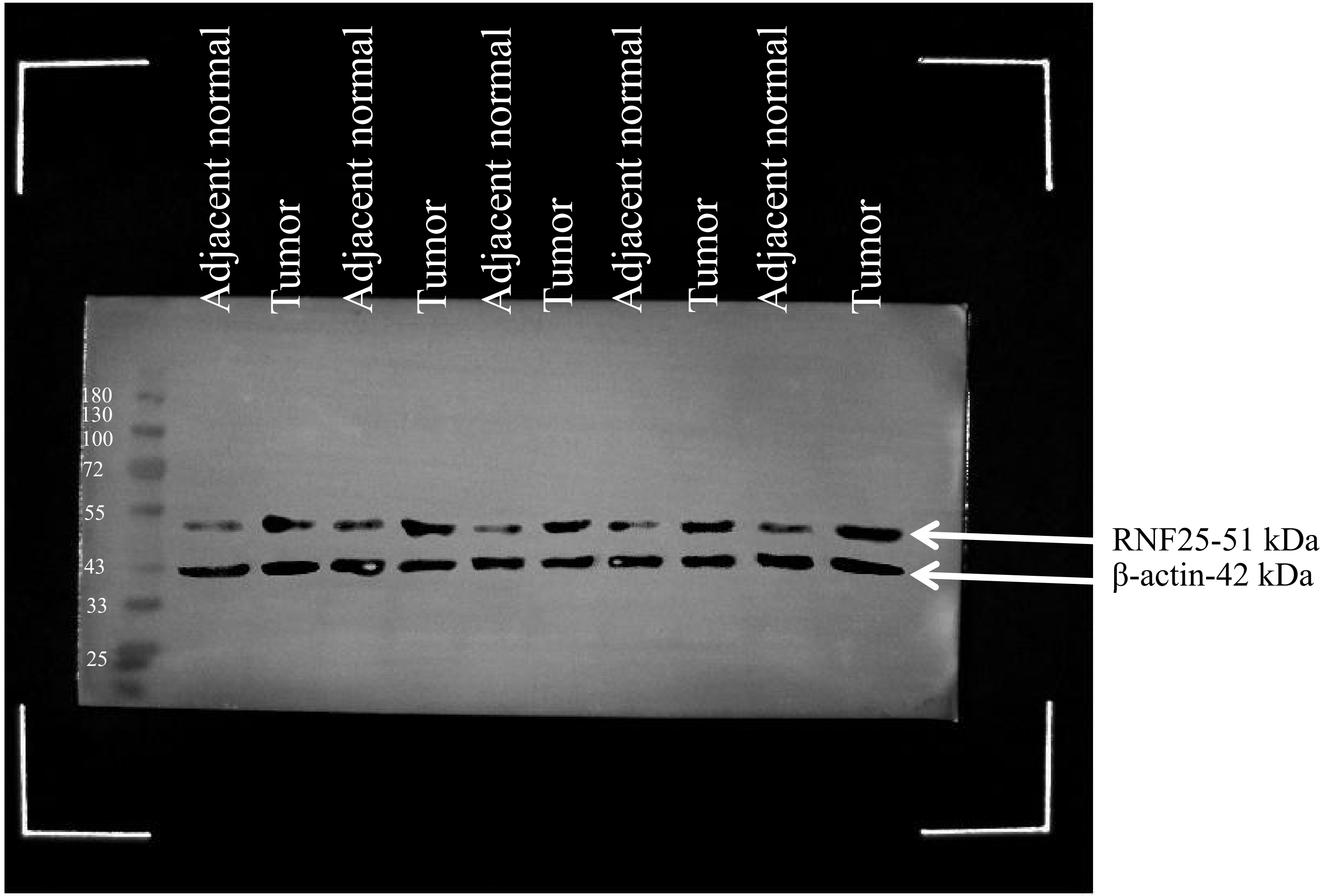
Adjacent normal
Tumor
Adjacent normal
Tumor
Adjacent normal
Tumor
Adjacent normal
Tumor
Adjacent normal
Tumor
180
130
100
72
55
43
33
25
RNF25-51 kDa
β-actin-42 kDa

Fig. 3E

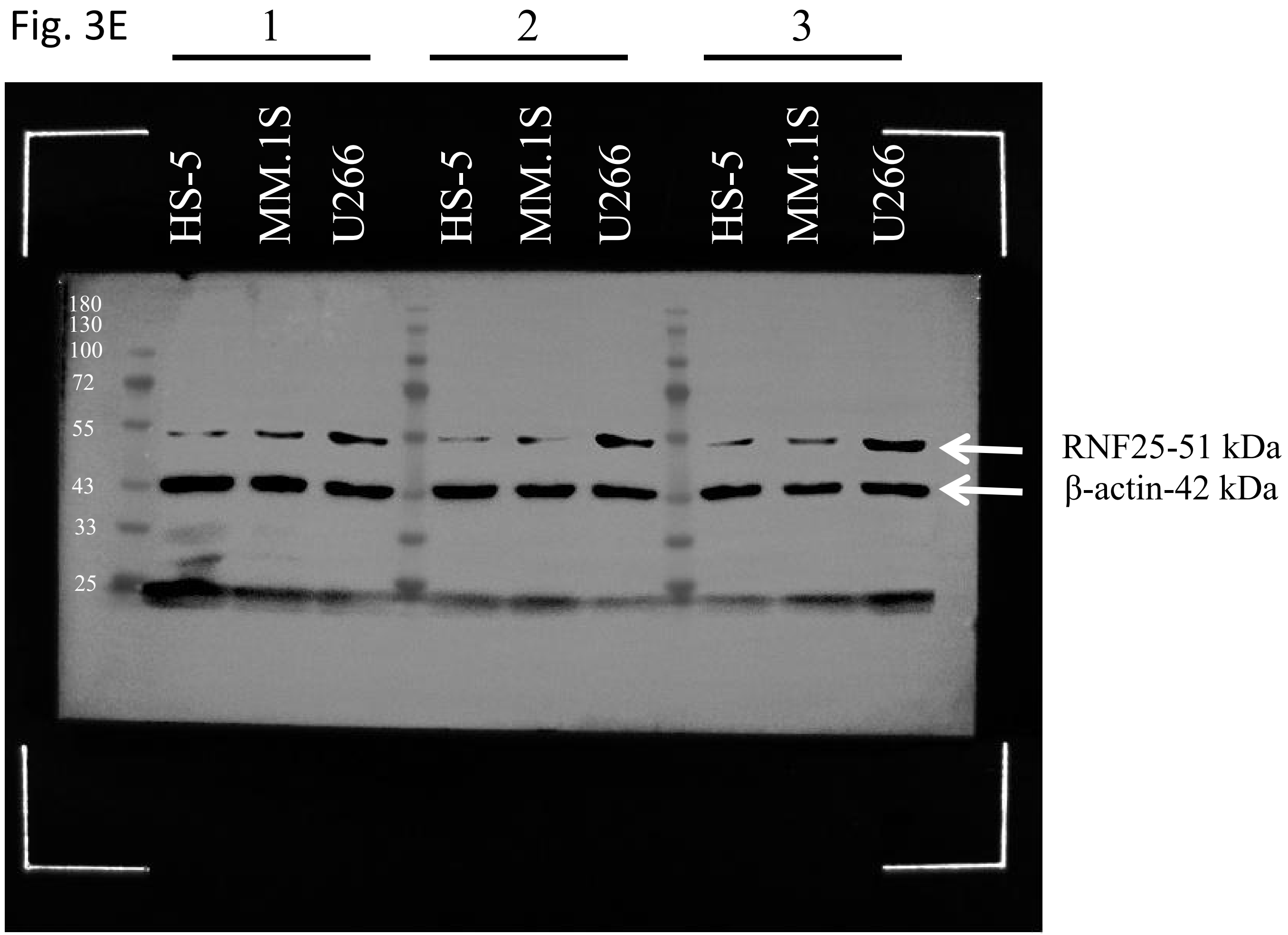

1
2
3
HS-5
MM.1S
U266
HS-5
MM.1S
U266
HS-5
MM.1S
U266
180
130
100
72
55
43
33
25
RNF25-51 kDa
β-actin-42 kDa

Fig. 9B

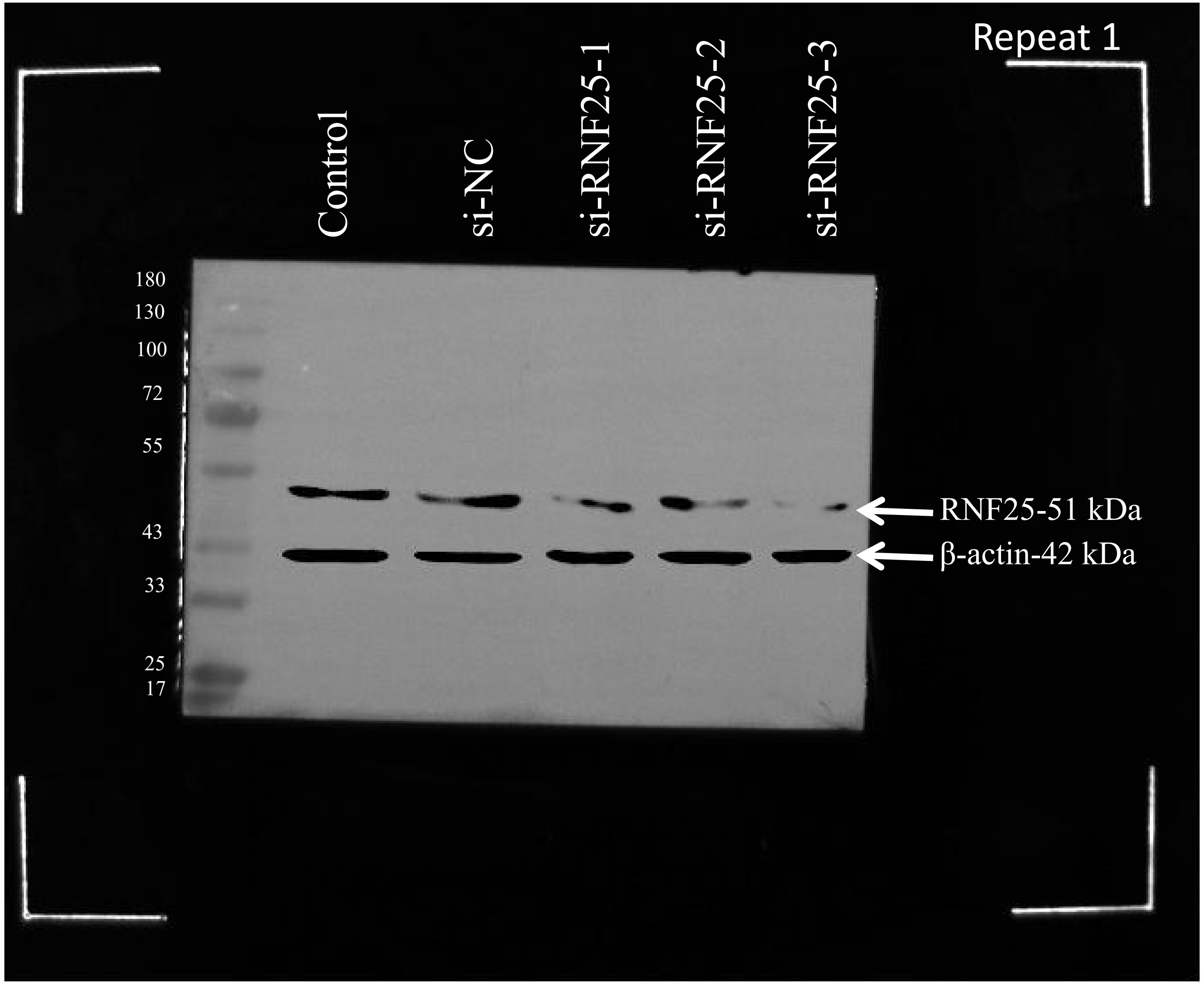
Repeat 1
Control
si-NC
si-RNF25-1
si-RNF25-2
si-RNF25-3
180
130
100
72
55
43
33
25
17
RNF25-51 kDa
β-actin-42 kDa

Fig. 9B

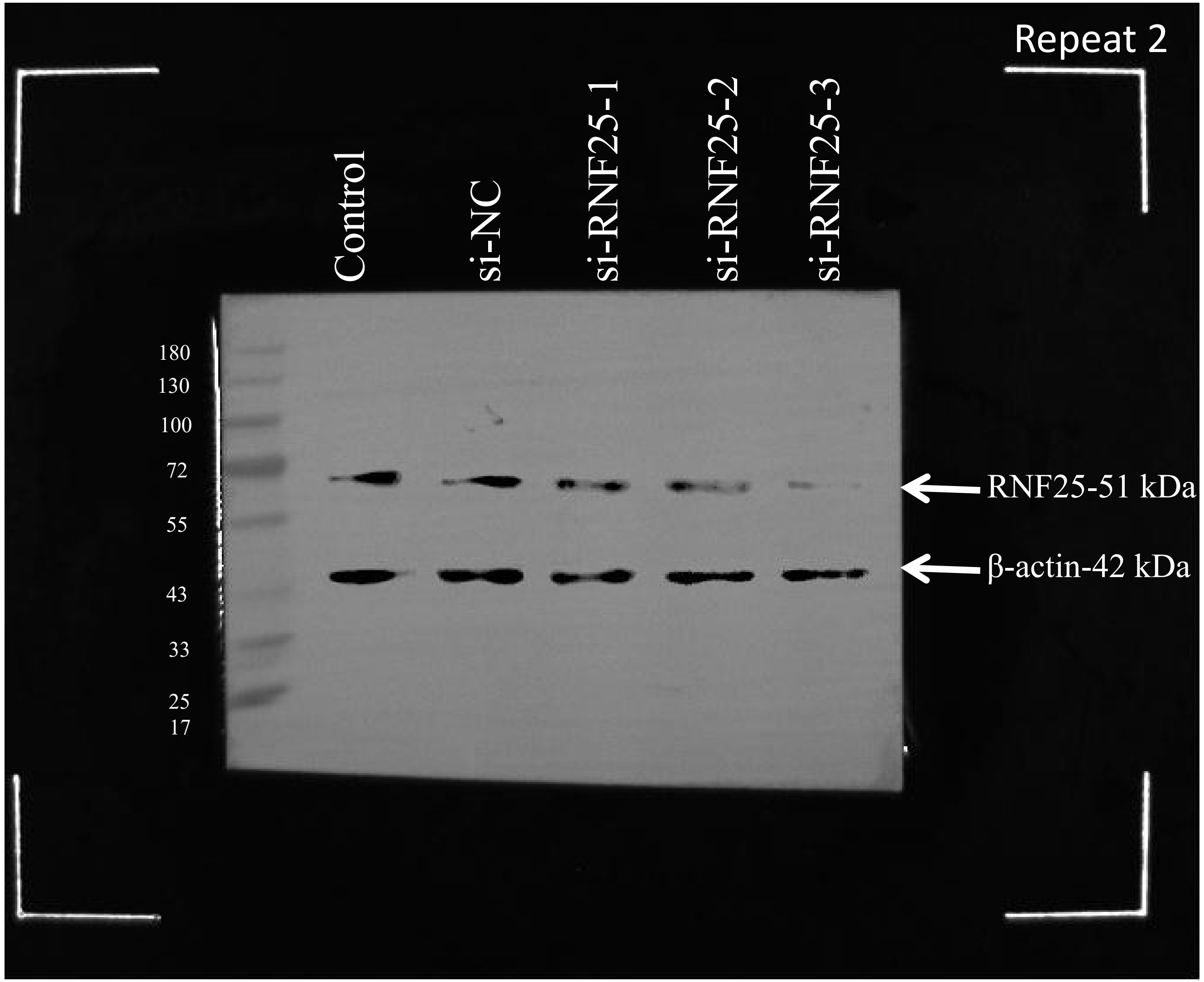
Repeat 2
Control
si-NC
si-RNF25-1
si-RNF25-2
si-RNF25-3
180
130
100
72
55
43
33
25
17
RNF25-51 kDa
β-actin-42 kDa

Fig. 9B

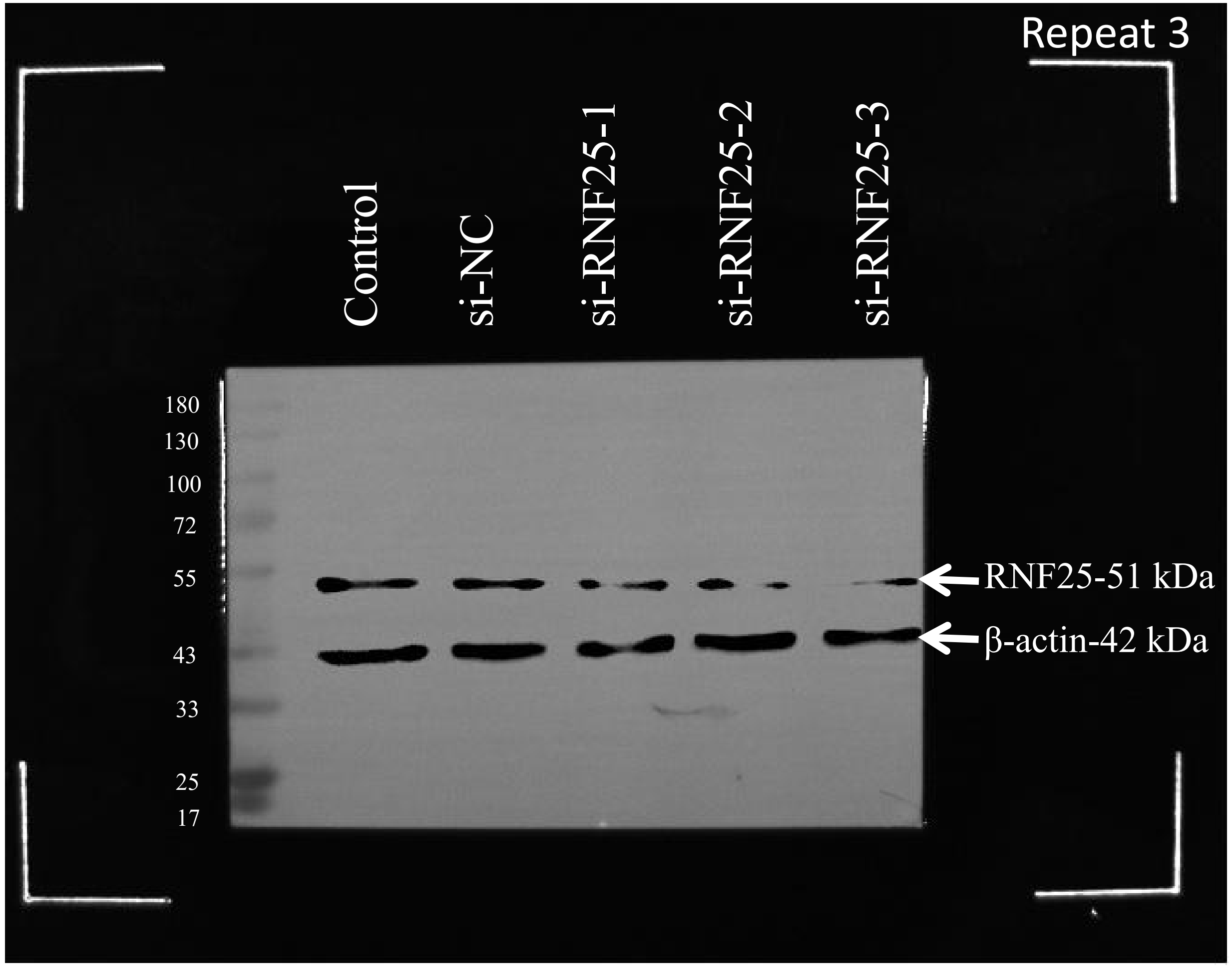
Repeat 3
Control
si-NC
si-RNF25-1
si-RNF25-2
si-RNF25-3
180
130
100
72
55
43
33
25
17
RNF25-51 kDa
β-actin-42 kDa
